# Ambiguity-Aware Part Annotation Refinement via Ontology Constraints

**DOI:** 10.3390/s26217056

**Published:** 2026-11-08

**Authors:** Seung Lee, Sukhan Lee, Juneho Yi

**Affiliations:** 1 Department of Electronic and Electrical Engineering, Sungkyunkwan University, Suwon 16419, Republic of Korea; mdibt@skku.edu; 2 Department of AI & Robotics, Sejong University, 209 Neungdong-ro, Gwangjin-gu, Seoul 05006, Republic of Korea; 3 Department of Electrical and Computer Engineering, Sungkyunkwan University, Suwon 16419, Republic of Korea; jhyi@skku.edu

**Keywords:** 3D part segmentation, semantic ambiguity, annotation refinement, ontology constraints, semantic interpretation, structural consistency, ShapeNet Part

## Abstract

Part annotation of 3D objects is important for part-level analysis, but local geometric evidence may not determine a unique semantic label. We propose an ontology-based annotation refinement framework that represents multiple semantic interpretations and filters candidates using structural and spatial constraints. For each point, a semantic interpretation set is constructed from labels observed in its local neighborhood, and ontology constraints yield a feasible subset without introducing new labels or forcing a single interpretation. We consider boundary ambiguity, part-definition ambiguity, and label-set incompleteness and evaluate the framework on the Airplane, Knife, and Skateboard categories of ShapeNet Part. Since feasible sets are subsets of semantic sets by construction, reductions in Ambiguity Size (AS) and Multi-label Ratio (MLR) are inherent properties of filtering, not independent evidence of improved annotation accuracy. The macro-average Ontology Filtering Rate (OFR) is 0.0947. Point-level analysis distinguishes unchanged candidate sets, partial filtering, and empty feasible sets, characterizing how the adopted ontology constraints affect candidate feasibility. The framework supports ambiguity-aware assessment of existing part annotations while preserving feasible alternatives, without claiming to resolve semantic ambiguity or recover ground-truth labels.

## 1. Introduction

### 1.1. Research Background

Recent advances in 3D sensing and deep learning have increased the importance of point clouds and 3D shape data for understanding object geometry and structure. The 3D Part Segmentation assigns semantic part labels to individual points, enabling detailed representations of object components. Early approaches such as PointNet demonstrated the feasibility of directly learning point-wise representations for 3D shape understanding and part segmentation [1]. Such representations support applications including robotic manipulation, 3D scene understanding, shape retrieval, and CAD model editing.

Point-level annotations provide supervision for segmentation models and references for performance evaluation, making annotation consistency important for reliable learning.

However, annotation differences do not necessarily indicate errors. The semantic interpretation of points near part boundaries may be uncertain, part definitions may admit different decompositions, and predefined label vocabularies may not represent all distinguishable components. Studies on fine-grained and multi-granularity part annotations also illustrate the variability of part definitions and decomposition levels [2,3]. These observations motivate distinguishing annotation ambiguity from annotation error in 3D part annotation.

**Prevailing Perspective on Annotation Inconsistency.** Dataset curation, noisy-label learning, and label correction generally seek to detect incorrect annotations or mitigate their effects during learning [4,5,6]. However, treating all annotation differences as errors may discard plausible interpretations arising from uncertain part boundaries, alternative definitions, or incomplete label vocabularies. This motivates evaluating structural and spatial compatibility while preserving locally observed alternatives.

### 1.2. Annotation Error and Annotation Ambiguity

In this study, annotation error refers to a labeling decision that is inconsistent with the intended semantic definition under the adopted annotation scheme. In contrast, annotation ambiguity arises when the available geometric evidence, part definitions, or label representation does not uniquely determine a semantic interpretation.

Different interpretations do not necessarily indicate annotation errors. For example, points near part interfaces may admit multiple plausible interpretations, while different part decomposition criteria may also affect semantic assignments [2]. Yi et al. [7] noted that multiple labeling solutions can be equally plausible for some parts. Accordingly, the proposed approach preserves possible interpretations and evaluates their feasibility using structural and spatial constraints rather than assuming that ambiguity must always be resolved into a single label.

**Research Problem.** Given an existing single-label 3D part annotation, the problem addressed in this study is how to represent locally observed alternative part interpretations and assess their compatibility with an explicitly adopted structural and spatial ontology without treating every disagreement as an annotation error. We therefore formulate refinement as candidate-set filtering, rather than prediction of a corrected ground-truth label: local annotation context defines the semantic candidates, and ontology constraints determine which of those candidates remain feasible. The resulting feasible set may contain multiple labels or be empty; neither outcome establishes the correctness of the original annotation.

### 1.3. Contributions

The main contributions of this study are as follows.

First, we distinguish annotation ambiguity from annotation error and label noise in 3D Part Annotation. Annotation ambiguity is considered in cases where the available geometry and labeling scheme do not uniquely determine a semantic interpretation.

Second, we characterize boundary ambiguity, part definition ambiguity, and label set incompleteness as representative sources of annotation ambiguity associated with geometry, semantic decomposition, and label representation, respectively. These sources motivate the representation of multiple semantic interpretations rather than serving as separate ambiguity-detection modules.

Third, we propose an ambiguity-aware annotation refinement framework that constructs a Semantic Interpretation Set and selectively removes candidates incompatible with ontology-defined structural and spatial constraints. The refinement process is formulated as

$$L_{\mathrm{sem}}\left(x_{i}\right)\to L_{\mathrm{feas}}\left(x_{i}\right)$$

where feasible semantic alternatives are preserved without introducing new candidate labels.

Fourth, we evaluate the framework on the Airplane, Knife, and Skateboard categories of ShapeNet Part. We analyze ontology-based candidate filtering using Ambiguity Size (AS), Multi-label Ratio (MLR), Ontology Filtering Rate (OFR), and complementary point-level measures. Because the feasible set is a subset of the semantic set, non-increasing AS and MLR values are inherent properties of the filtering process rather than independent empirical findings. The experiments quantify the extent of filtering and examine its category-specific behavior through quantitative and qualitative analyses.

## 2. Related Work

### 2.1. 3D Part Segmentation Datasets and Annotation

ShapeNet is a widely used 3D shape repository containing CAD models from diverse object categories [8]. Built upon ShapeNet, ShapeNet Part provides point-level semantic part annotations and serves as a representative benchmark for 3D part segmentation [7].

Representative methods, including PointNet [1], PointNet++ [9], Dynamic Graph CNN (DGCNN) [10], and Point Transformer [11], have used ShapeNet Part for training and evaluation. Recent approaches have also explored global and local feature representations to improve part segmentation performance [12].

PartNet provides a large-scale benchmark for fine-grained and hierarchical part-level 3D object understanding [2]. Its hierarchical part annotations illustrate that part representations may differ in their granularity and decomposition structure. Related work on multi-granularity open-vocabulary part segmentation has also investigated the interpretation of parts at different semantic granularities [3].

ShapeNet Part adopts a single-label annotation scheme in which each point is assigned one predefined semantic part label. Although this representation provides explicit supervision and supports standardized evaluation, it does not necessarily imply that each point admits only one possible semantic interpretation. Ambiguity, variation, uncertainty, and error have been discussed as distinct annotation phenomena [13], and disagreements between annotators do not necessarily indicate annotation errors [14].

These observations motivate the candidate-set formulation introduced in Section 3.

### 2.2. Annotation Refinement and Learning from Ambiguous Annotations

Annotation refinement and noisy-label learning have been widely studied to address errors and inconsistencies in annotated datasets. Existing approaches include confidence estimation, sample selection, and label correction, primarily aiming to identify unreliable annotations or mitigate their effects during learning [4,5,6]. In 3D point cloud segmentation, noise-robust learning methods have also exploited point-wise information and structural characteristics to reduce the influence of noisy annotations [15,16].

Confident Learning identifies potential label errors by analyzing relationships between observed labels and model-predicted class probabilities [4], while Frénay and Verleysen [6] provide a broader review of learning under label noise. These approaches contribute to annotation reliability, but correcting an unreliable label is not equivalent to evaluating multiple plausible semantic interpretations. In particular, annotation disagreement does not necessarily indicate an error, and enforcing a single corrected label may discard plausible alternatives.

Partial Label Learning (PLL) considers multiple candidate labels associated with an instance [17,18]. Conventional PLL generally assumes that the candidate set contains a latent ground-truth label and aims to identify that label. Candidate Label Set Pruning (CLSP) further investigates candidate removal based on inconsistencies between representation and candidate-label spaces [19]. In contrast, the Semantic Interpretation Set used in this study represents locally observed alternatives without requiring that only one candidate be semantically valid.

Unlike label correction or single-label disambiguation, our approach evaluates candidate feasibility without requiring a unique label.

### 2.3. Ontology-Based Knowledge Representation for 3D Shape Understanding

Ontologies explicitly represent concepts, properties, and relationships through structured knowledge and logical constraints [20]. In 3D shape understanding, ontology-based approaches have been used to represent semantic concepts, object structures, and spatial relationships [21,22]. Spatial and topological relationships can also be represented and evaluated through ontology-based reasoning mechanisms [23].

Structural, functional, and spatial relationships among object parts provide knowledge for describing their semantic organization [24,25,26]. Previous studies have investigated semantic shape annotation, part relationships, and consistent segmentation [27,28]. Ontology-based 3D segmentation has demonstrated the use of domain knowledge and spatial relationships [29], while geometric and spatial information has been used to extract spatial semantics from point clouds [30].

Object-level structural knowledge can constrain locally observed candidates beyond geometric evidence alone.

Representative approaches addressing annotation refinement, candidate pruning, ontology-based representation, and spatial reasoning are compared in Table 1.

These approaches differ in their objectives and output representations. Unlike methods targeting label correction or unique-label prediction, the proposed framework filters local semantic candidates using ontology constraints without claiming superior segmentation accuracy.

### 2.4. Research Gap

Prior work provides foundations in segmentation, noisy-label learning, partial-label learning, and ontology-based reasoning [4,5,6,7,15,17,18,20,21,22,23,24,25,26,27,29,30], but two gaps remain.

First, existing annotation refinement and noisy-label learning methods primarily address annotation inconsistency as error or noise, while conventional PLL generally seeks to identify a latent ground-truth label from multiple candidates. Research on fine-grained and multi-granularity part understanding also demonstrates that part representations can vary according to the adopted decomposition and granularity [2,3]. However, these approaches do not directly provide the specific candidate-preserving, ontology-constrained refinement procedure considered in this study.

Second, ontology-based approaches provide explicit representations of semantic, structural, and spatial relationships. However, their use as feasibility constraints for selectively filtering multiple semantic interpretation candidates, rather than directly predicting a unique label, remains relatively underexplored. This motivates combining local semantic alternatives with object-level structural and spatial compatibility assessment.

These research gaps concern differences in problem formulation and methodological objectives rather than establishing performance superiority. In particular, the proposed framework evaluates structural and spatial compatibility among existing annotation candidates without assuming that every ambiguity corresponds to a label error or must be resolved into a single corrected label.

The following section presents the conceptual framework for ontology-constrained filtering of locally observed semantic alternatives.

## 3. Conceptual Framework for Annotation Ambiguity

### 3.1. Annotation Ambiguity as Semantic Interpretation Set

Annotation ambiguity can arise from different aspects of 3D Part Annotation. In this study, we consider boundary ambiguity, part definition ambiguity, and label set incompleteness as representative sources associated with geometry, semantic decomposition, and label representation, respectively. These sources are not intended as an exhaustive classification or as separate ambiguity-detection mechanisms. Previous studies on fine-grained part annotation provide relevant evidence of variation in part decomposition and semantic interpretation [2].

First, *boundary ambiguity* arises when the geometric transition between adjacent parts does not clearly determine a unique semantic assignment. For example, points near the body–wing interface of an airplane may admit different plausible part interpretations without necessarily indicating annotation errors.

Second, *part definition ambiguity* arises when the semantic extent or composition of a part depends on the adopted part definition or annotation criteria. Different criteria for decomposing an object into semantic parts may lead to different interpretations of similar structures [2].

**Figure 1:**
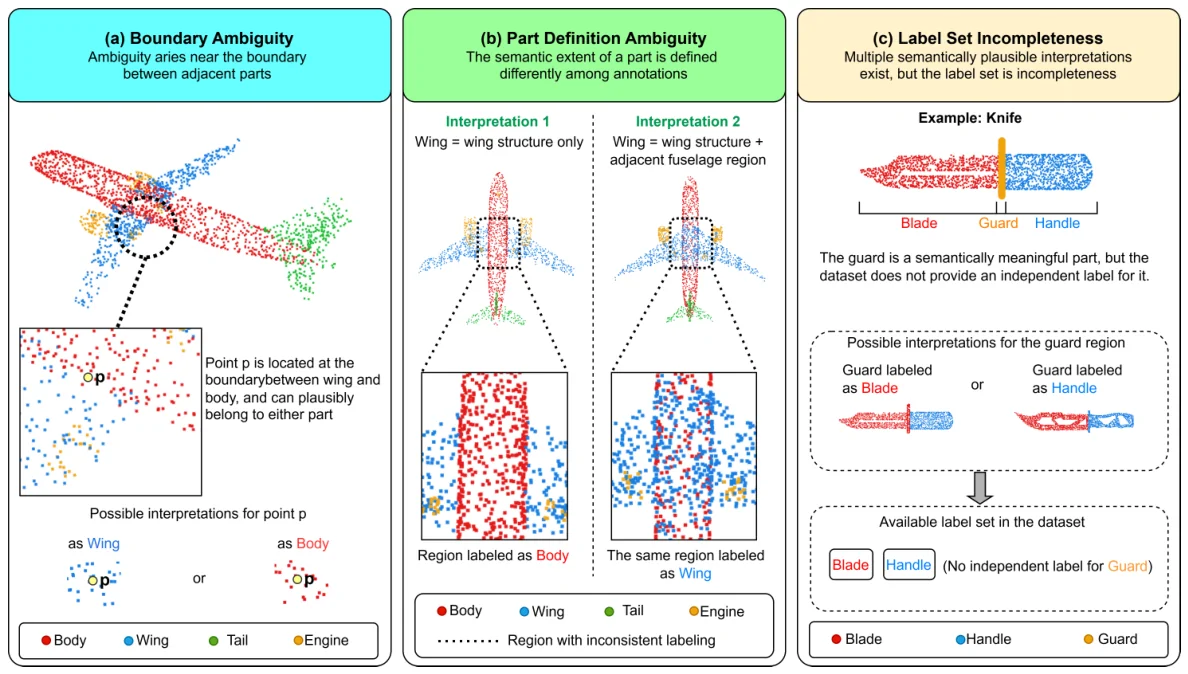
Representative sources of annotation ambiguity in 3D part annotation: (**a**) boundary ambiguity; (**b**) part definition ambiguity, illustrated by two observed Wing annotation configurations with different spatial extents in ShapeNet Part; and (**c**) label set incompleteness.

Figure 1b presents two Wing annotation configurations observed in ShapeNet Part. In one case, the Wing region is annotated on the left and right sides of the Body, whereas in the other case, the annotated Wing region extends across the central body region as a single connected region. These observations illustrate variations in the annotated spatial extent of the Wing part, which may reflect differences in part definition or annotation criteria rather than necessarily indicating annotation errors.

The ontology adopted in this study represents one specific Wing definition, in which Wing candidates are evaluated according to their left/right spatial relationships with respect to the Body reference part. Therefore, the adopted ontology does not necessarily accommodate all Wing annotation configurations observed in ShapeNet Part. An interpretation that violates these constraints is considered infeasible under the adopted ontology, but this does not necessarily imply that the original annotation is erroneous. Accordingly, the proposed framework preserves alternative interpretations only when they satisfy the selected ontology constraints; it does not simultaneously preserve interpretations arising from different, potentially incompatible part definitions.

Third, *label set incompleteness* arises when the available annotation vocabulary lacks an independent label for a structurally distinguishable component. For example, a knife guard is structurally distinguishable from the blade and handle, but ShapeNet Part does not provide an independent Guard label [7]. Consequently, the guard cannot be represented as an independent semantic part under the adopted label vocabulary. This limitation does not necessarily indicate an annotation error.

These three sources illustrate different reasons why semantic interpretation may not be uniquely determined. In the proposed framework, they serve as conceptual motivations rather than separate ambiguity-detection procedures. The framework operationalizes locally observed semantic alternatives through the Semantic Interpretation Set defined below.

Representative sources of annotation ambiguity are illustrated in Figure 1.

For each point $x_{i}$, the Semantic Interpretation Set $L_{\mathrm{sem}}\left(x_{i}\right)$ is defined as the set of distinct part labels observed in its local neighborhood, including the original annotation of $x_{i}$. This provides an operational representation of locally observed semantic alternatives without assuming that all candidates are equally valid or that a unique ground-truth label must be recovered.

The neighborhood construction and its parameter settings are described in Section 4.

### 3.2. Our Perspective

From the perspective of annotation ambiguity, refinement should not necessarily force every point to a unique semantic interpretation. Instead, plausible alternatives should be preserved, while candidates incompatible with the structural and spatial organization of the object may be removed. Because part annotations collectively represent object structure, their consistency depends on relationships among parts as well as individual point interpretations [27,28].

The Semantic Interpretation Set represents locally observed alternatives, but local evidence alone does not establish their compatibility with object-level relationships. To incorporate such knowledge, we use a category-specific ontology to represent structural and spatial relationships among parts and formulate corresponding feasibility constraints [27,29]. The adopted ontology reflects a particular part definition and object configuration rather than a universal representation of all valid interpretations.

Ontology-based infeasibility therefore indicates incompatibility with the adopted constraints, not necessarily an annotation error. The ontology neither introduces new labels nor determines a unique ground-truth interpretation.

### 3.3. Ontology-Constrained Refinement Framework

For each point $x_{i}$, the framework first constructs a Semantic Interpretation Set $L_{\mathrm{sem}}\left(x_{i}\right)$ from the local annotation context. Ontology constraints are then applied to remove candidates that are incompatible with the adopted structural and spatial relationships. The remaining candidates constitute the Feasible Interpretation Set $L_{\mathrm{feas}}\left(x_{i}\right)$, which satisfies $L_{\mathrm{feas}}\left(x_{i}\right)\subseteq L_{\mathrm{sem}}\left(x_{i}\right)$.

The overall refinement process is expressed as

(1) $$A\left(x_{i}\right)\to L_{\mathrm{sem}}\left(x_{i}\right)\to L_{\mathrm{feas}}\left(x_{i}\right)$$

Here, $A(x_{i})$ denotes the original annotation, $L_{\mathrm{sem}}\left(x_{i}\right)$ denotes locally observed semantic alternatives, and $L_{\mathrm{feas}}\left(x_{i}\right)$ denotes the candidates retained after ontology-based feasibility evaluation.

**Figure 2:**
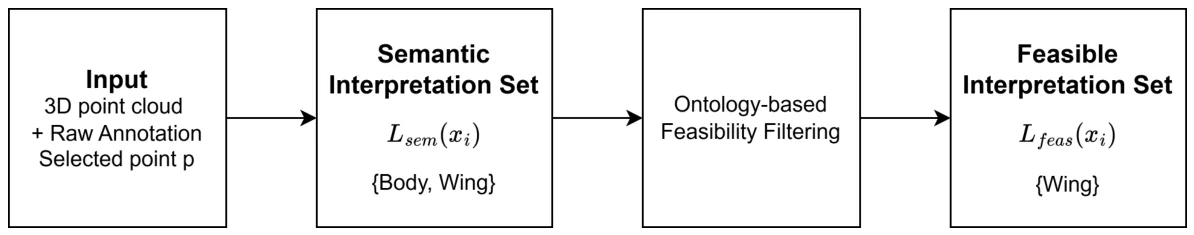
Conceptual overview of the proposed ambiguity-aware annotation refinement framework. The framework first constructs a semantic interpretation set ($L_{\mathrm{sem}}\left(x_{i}\right)$) and then applies ontology constraints to remove only structurally and spatially infeasible interpretations, producing a feasible interpretation set ($L_{\mathrm{feas}}\left(x_{i}\right)$).

Figure 2 illustrates the conceptual flow of the proposed framework.

The following section describes the methodological procedures for constructing the Semantic Interpretation Set and applying ontology-based feasibility refinement.

## 4. Methods

**Figure 3:**
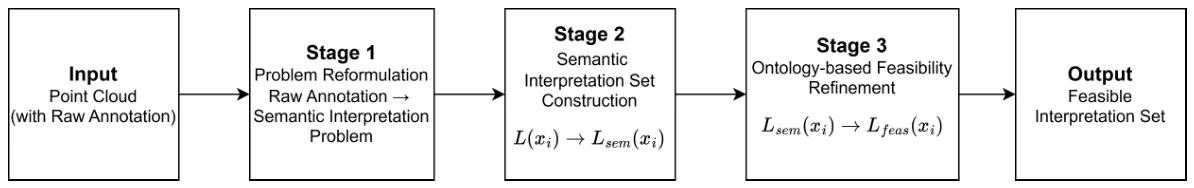
Overview of the proposed framework. The framework consists of (1) problem formulation, (2) semantic interpretation set construction, where observed annotation labels are collected from the local neighborhood of each point, and (3) ontology-based feasibility refinement, where incompatible candidates are filtered according to structural and spatial constraints. The framework preserves feasible semantic alternatives without performing direct label prediction or annotation correction.

Figure 3 summarizes the implementation stages: problem formulation, neighborhood-based semantic candidate construction, and ontology-based feasibility evaluation. The following subsections specify these procedures.

### 4.1. Problem Definition

We formulate annotation refinement as filtering semantic candidates rather than assigning a corrected label. Starting from the existing annotation, the method retains locally observed interpretations unless they are incompatible with the applicable structural and spatial constraints.

Let a point cloud be represented as

(2) $$P={\left\{x_{i}\right\}}_{i=1}^{N},$$

where $x_{i}$ denotes the *i*-th point and *N* denotes the total number of points. The set of predefined part labels for the object category is defined as

(3) $$L=\{l_{1},l_{2},\ldots ,l_{M}\},$$

where *M* denotes the total number of part labels considered for the object category. The existing annotation of the input point cloud is represented by the following mapping:

(4) A:P→L,

where $A(x_{i})$ denotes the label originally assigned to point $x_{i}$. Importantly, $A(x_{i})$ is treated as the observed annotation and is not assumed to represent the only plausible semantic interpretation of $x_{i}$.

A semantic interpretation set is then constructed from the local neighborhood of each point as follows:

(5) $$L_{\mathrm{sem}}\left(x_{i}\right)=\Psi_{\mathrm{sem}}\left(x_{i}\right)\subseteq L,$$

where $\Psi_{\mathrm{sem}}$ denotes the process of collecting observed annotation labels from the local neighborhood of $x_{i}$. In this study, $x_{i}$ itself is included in the local neighborhood, so at least one annotation label is always observed within the neighborhood. Therefore, the following condition holds:

(6) $$|L_{\mathrm{sem}}\left(x_{i}\right)|\geq 1.$$

For example, if both *Wing*- and *Body*-annotated points are present around a point in an airplane, the semantic interpretation set can be represented as $L_{\mathrm{sem}}\left(x_{i}\right)=\left\{\mathrm{Wing},\mathrm{Body}\right\}$. At this stage, the co-occurrence of the two labels is not used to determine either label as an annotation error. Instead, both labels are retained as candidate interpretations for subsequent feasibility evaluation.

The feasible interpretation set is then defined using category-specific structural and spatial constraints as follows:

(7) $$L_{\mathrm{feas}}\left(x_{i}\right)=\Psi_{\mathrm{feas}}\left(x_{i}\right)\subseteq L_{\mathrm{sem}}\left(x_{i}\right),$$

where $\Psi_{\mathrm{feas}}$ denotes the feasibility filtering process that removes candidate interpretations incompatible with the applicable structural and spatial constraints. Thus, the overall refinement process can be expressed as

(8) $$A\left(x_{i}\right)\to L_{\mathrm{sem}}\left(x_{i}\right)\to L_{\mathrm{feas}}\left(x_{i}\right).$$

**Figure 4:**
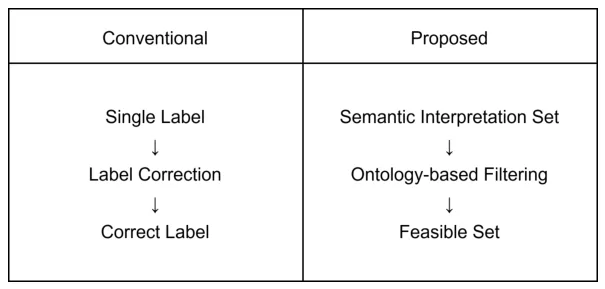
Comparison between the conventional annotation refinement paradigm and the proposed ambiguity-aware formulation. Conventional annotation refinement typically formulates the task as recovering a single semantic label for each point through label correction. In contrast, the proposed framework represents multiple locally observed semantic alternatives using a semantic interpretation set and refines them through ontology-based constraint filtering to obtain the feasible interpretation set while preserving compatible ambiguity.

Importantly, $L_{\mathrm{feas}}\left(x_{i}\right)$ is not required to contain a single label. When multiple interpretations satisfy the applicable constraints, multiple candidates are retained. Conversely, if none of the candidates in $L_{\mathrm{sem}}\left(x_{i}\right)$ satisfies the constraints, $L_{\mathrm{feas}}\left(x_{i}\right)=\emptyset$ may result. The empty set indicates that no feasible interpretation was found among the currently available candidates; it does not trigger the generation or inference of a new semantic label. The proposed formulation therefore preserves candidate interpretations that remain compatible with the category-specific constraints while removing candidates that are explicitly incompatible with them. The proposed ambiguity-aware formulation is illustrated in comparison with the conventional annotation refinement paradigm in Figure 4.

### 4.2. Semantic Interpretation Set Construction

The Semantic Interpretation Set is constructed from the annotation labels observed in the local geometric neighborhood of each point. For a point $x_{i}$, its neighborhood is defined using the *k* nearest neighbors in the 3D coordinate space:

(9) $$N_{k}\left(x_{i}\right)=\left\{x_{j}|x_{j}\;\mathrm{is}\;\mathrm{one}\;\mathrm{of}\;\mathrm{the}\;k\;\mathrm{nearest}\;\mathrm{neighbors}\;\mathrm{of}\;x_{i}\right\}.$$

The query point $x_{i}$ itself is included in $N_{k}\left(x_{i}\right)$. In this study, k=20 is used in all experiments, so each neighborhood consists of the query point and its 19 nearest neighbors. The neighborhood size k=20 was empirically selected based on preliminary trials with different neighborhood sizes and visual inspection of the resulting candidate distributions. This value was consistently used across all evaluated categories. No formal cross-validation or systematic sensitivity analysis was performed, and the selected value is not claimed to be optimal.

The Semantic Interpretation Set is constructed by collecting the annotation labels observed within the neighborhood:

(10) $$L_{\mathrm{sem}}\left(x_{i}\right)=\left\{A\left(x_{j}\right)|x_{j}\in N_{k}\left(x_{i}\right)\right\}.$$

Because the result is represented as a set, duplicate labels are automatically removed. All distinct labels observed in the neighborhood are retained as candidate interpretations, without majority voting or representative-label selection.

The construction procedure is summarized in Algorithm 1.

For example, a point near the Wing–Body boundary of an Airplane may have both Wing and Body labels within its local neighborhood. In this case, its interpretation set becomes $L_{\mathrm{sem}}\left(x_{i}\right)=\left\{\mathrm{Wing},\mathrm{Body}\right\}$. The co-occurrence of these labels does not by itself determine either interpretation to be erroneous. Instead, both candidates are retained for subsequent ontology-based feasibility evaluation.

The same construction can retain locally observed alternatives in regions where the semantic extent of a part may admit different interpretations. However, the procedure does not introduce labels that are absent from the original annotation vocabulary. For example, the Knife guard is not assigned an independent label in ShapeNet Part and is therefore not introduced as a candidate in $L_{\mathrm{sem}}\left(x_{i}\right)$. Its geometric information is detected separately and used as an auxiliary spatial cue during ontology-based refinement.
**Algorithm 1:** Semantic Interpretation Set Construction
 **Input**: Point cloud *P*; original annotations *A*; neighborhood size *k*

 **Output**: Semantic interpretation sets ${\left\{L_{\mathrm{sem}}\left(x_{i}\right)\right\}}_{i=1}^{N}$
 
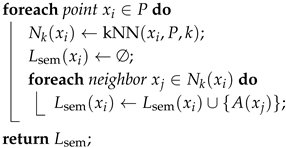



**Figure 5:**
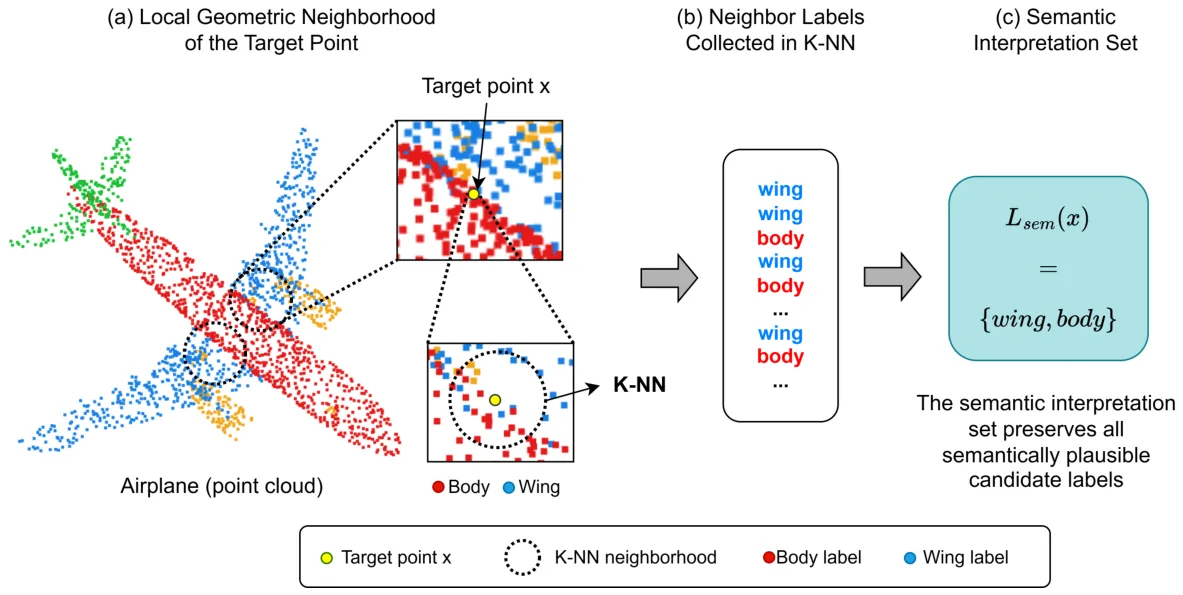
Illustration of the semantic interpretation set construction process. For each point, the *k*-nearest neighbors are identified in the local neighborhood, and the annotation labels of neighboring points are collected to form a unique candidate label set. Instead of selecting a single representative label, the proposed method preserves the observed candidate labels as the semantic interpretation set, representing locally observed semantic alternatives. The green dot marks the target point, orange dots denote another part category in the example, and the ellipses indicate omitted entries in the illustrated neighbor-label list.

Figure 5 illustrates the construction of the Semantic Interpretation Set from locally observed annotation labels.

The resulting Semantic Interpretation Set represents locally observed candidate labels rather than a corrected annotation or a predicted semantic label. Because local neighborhood information alone does not establish compatibility with object-level structural and spatial relationships, the candidate set may contain interpretations that violate the adopted ontology constraints. The following subsection describes how such candidates are evaluated through ontology-based feasibility refinement.

### 4.3. Ontology-Based Feasibility Refinement

The semantic interpretation set may contain candidate labels that are incompatible with the structural and spatial constraints applicable to the corresponding object category. In this study, ontology-based feasibility refinement (hereafter, ontology-based refinement) denotes the process of selectively removing such candidates from $L_{\mathrm{sem}}\left(x_{i}\right)$.

The refinement process does not generate new candidates or convert one candidate into another label. Instead, the candidates already contained in $L_{\mathrm{sem}}\left(x_{i}\right)$ are evaluated against the category-specific ontology constraints. Candidates that are explicitly incompatible with the applicable structural and spatial constraints are removed, whereas candidates for which no incompatibility is identified are retained.

**Figure 6:**
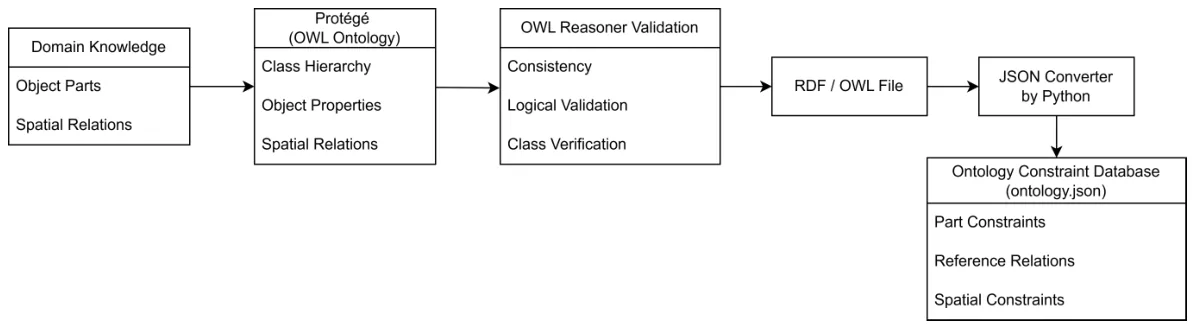
Ontology construction and conversion process. Domain knowledge is modeled in Protégé as an OWL ontology and validated using an OWL reasoner. The validated ontology is stored as an RDF/OWL file and converted into a JSON-based ontology constraint database (ontology.json) using a Python 3.12.9 converter, which is subsequently used for ontology-based refinement.

**Figure 7:**
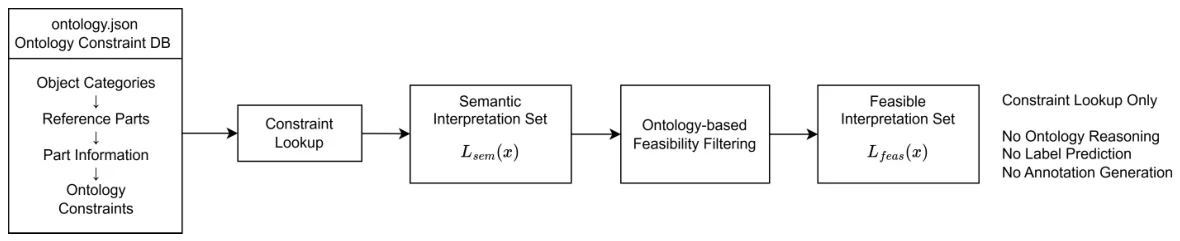
Implementation of the ontology constraint database used during refinement. The OWL ontology is transformed into a hierarchical JSON structure containing object categories, reference parts, part information, and ontology constraints. During refinement, ontology constraints are retrieved through constraint lookup without performing ontology reasoning, label prediction, or annotation generation.

**Figure 8:**
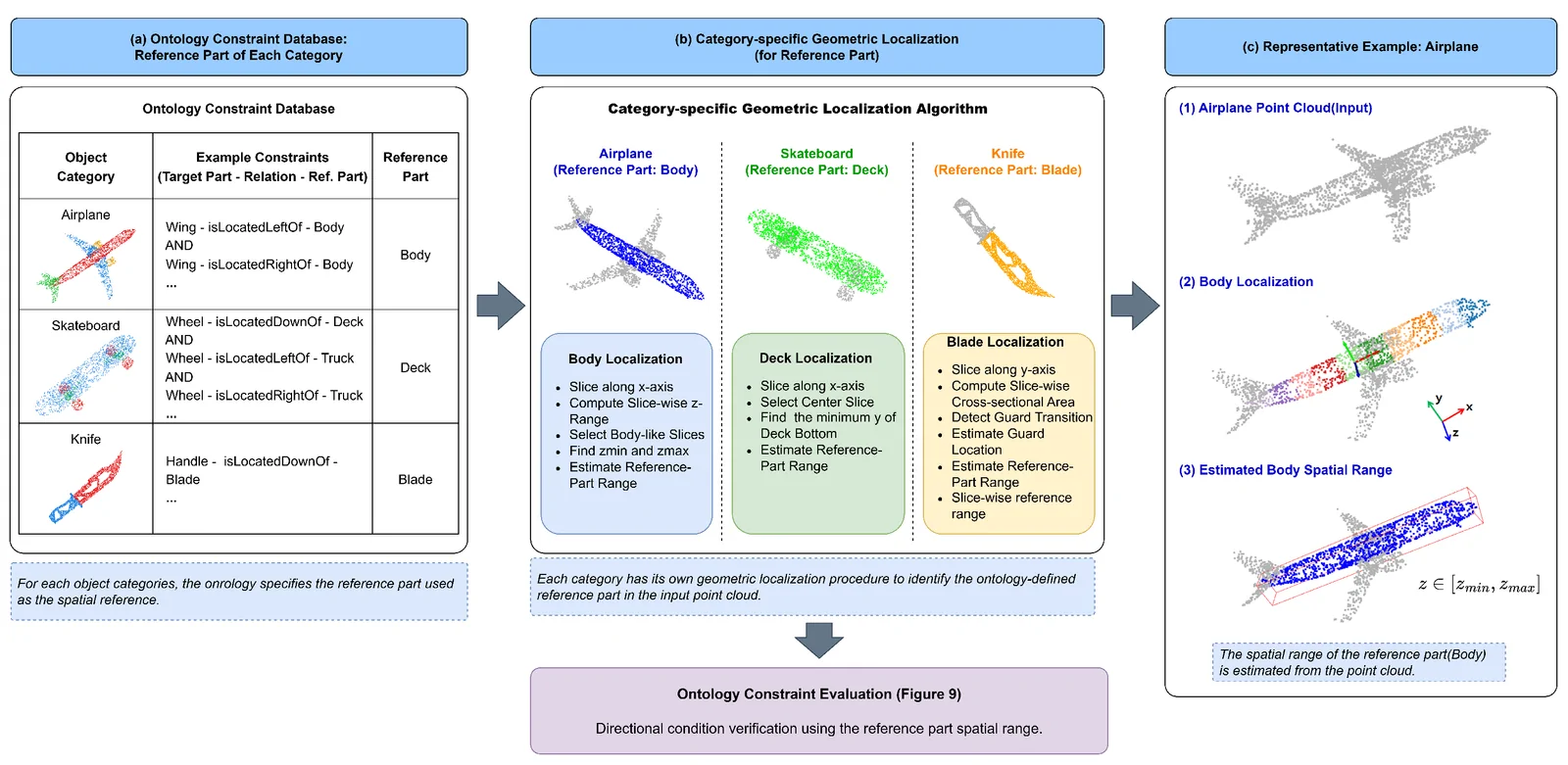
Reference part localization for each object category. The reference part is localized from the input point cloud using category-specific geometric and spatial characteristics. For Knife, the localized Blade is further used to estimate the slice-wise reference spatial range required for ontology constraint evaluation. The ellipses in (**a**) indicate that only representative ontology constraints are shown. Colors in (**a**) distinguish object parts, while those in (**b**) highlight the reference parts of each category. In (**c**), different colors in step (2) distinguish slices along the *x*-axis, and the blue points in step (3) indicate the localized Body points used to estimate its spatial range.

**Figure 9:**
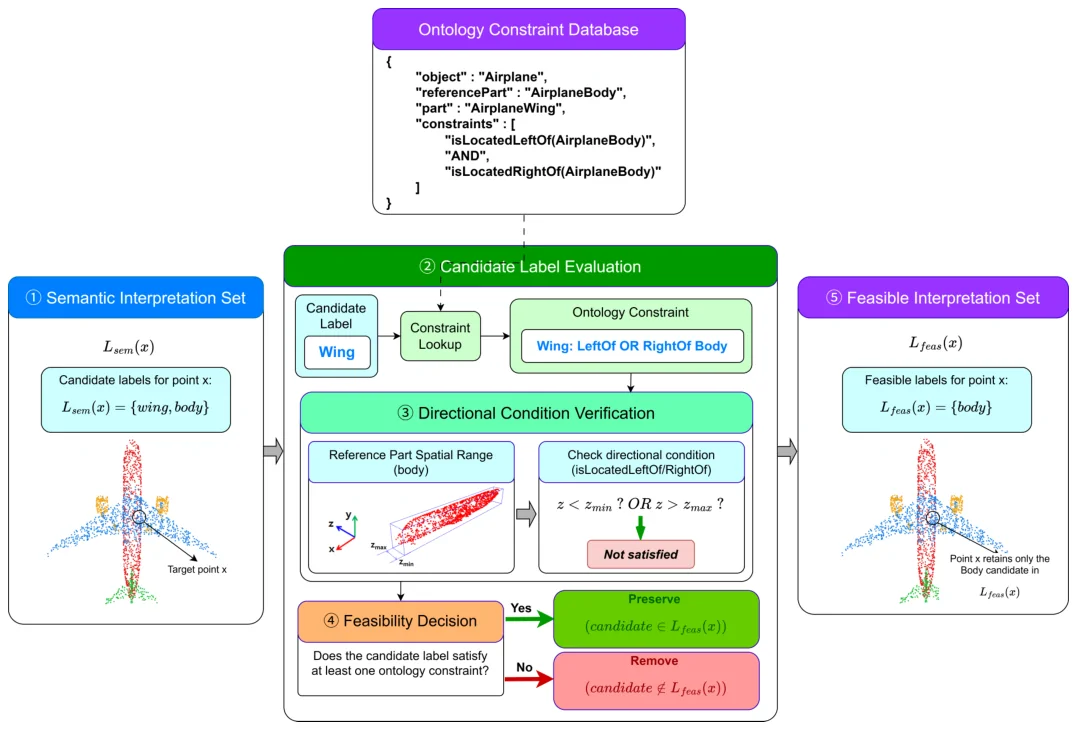
Ontology constraint evaluation for candidate filtering. For each candidate label, the corresponding ontology constraint is retrieved from the ontology constraint database and evaluated using the estimated reference part spatial range. Multiple directional conditions within a constraint are jointly evaluated, while alternative constraint conditions can be represented as disjunctive cases. The example illustrates the evaluation of the Airplane Wing candidate using the left/right spatial relations with respect to the Airplane Body. Colored blocks distinguish the input semantic interpretation set, candidate evaluation stages, and output feasible interpretation set; the green and red paths indicate candidate preservation and removal, respectively. The dashed arrow indicates the retrieval of ontology constraints from the ontology constraint database, and the question marks indicate the directional conditions checked against the spatial range of the reference part.

Ontology-based refinement is implemented through four procedures: (1) OWL-based ontology construction, (2) JSON-based ontology constraint database implementation, (3) reference part spatial range estimation, and (4) ontology constraint evaluation. These procedures describe the implementation of ontology-based refinement rather than additional semantic interpretation stages. Figure 6, Figure 7, Figure 8 and Figure 9 illustrate the overall process of these procedures.

**OWL-based Ontology Construction.** In this study, a category-specific ontology was constructed to represent the constituent parts and structural and spatial relationships of the selected object categories. Ontology-based representations have been used to describe functional and structural relationships in product and system design [24,25,26], as well as spatial relationships between components with respect to a reference component [30]. The ontology in this study is not intended to provide a universal definition of part semantics; rather, it encodes the relationships required for evaluating candidate interpretations under the adopted part decomposition. The ontology was implemented using the Web Ontology Language (OWL) [20] and Protégé [33].

For each object category, one reference part is defined as the spatial basis for evaluating the relative positions of other parts. Specifically, Body, Blade, and Deck are used as the reference parts for Airplane, Knife, and Skateboard, respectively. The reference part serves as a spatial reference for constraint evaluation rather than necessarily representing the overall functional component of the object.

Object-part relationships are represented using properties such as *hasPart*, *hasReferencePart*, and *hasFunction*. The *hasFunction* property describes functional relationships but is not used as a direct filtering condition. Spatial feasibility is evaluated using six directional relations: *isLocatedFrontOf*, *isLocatedBackOf*, *isLocatedLeftOf*, *isLocatedRightOf*, *isLocatedUpOf*, and *isLocatedDownOf*. These relations specify the relative positions of candidate parts with respect to the reference part.

For Knife, the guard is treated as a handle-side structure for modeling purposes because it is not represented as an independent semantic label in ShapeNet Part. This is a category-specific modeling convention rather than a universal definition of the guard. The guard is not introduced as a new candidate label; instead, its geometric characteristics are used as an auxiliary spatial cue for estimating the Blade reference range and evaluating spatial constraints.

The ontology was validated using an OWL reasoner and stored in RDF/OWL format. The reasoner was used only to verify the logical structure of the ontology, not to infer point labels or perform annotation refinement. The validated ontology was subsequently converted into a JSON-based constraint database for use in the refinement process, as illustrated in Figure 6.

The OWL/Protégé representation offers practical advantages in logical consistency checking, reusability, and extensibility. Common structural and directional relations can be reused across object categories, while additional part classes and category-specific constraints can be incorporated without changing the overall refinement procedure. However, extending the ontology to new categories requires appropriate structural knowledge and reference-part localization. These advantages concern ontology representation and management; point-level feasibility filtering is performed separately using the exported constraints.

**Ontology Constraint Database Implementation.** The validated RDF/OWL ontology is converted into a JSON-based constraint database for efficient retrieval of category-specific constraints during point-level refinement. The database stores the reference part, part information, and structural and spatial constraints for each object category. During refinement, the corresponding constraints are retrieved according to the object category and candidate label.

For each candidate label (*l*), the corresponding set of ontology constraints is denoted by (C(l)). A single constraint may contain multiple directional conditions, all of which must be satisfied simultaneously (AND). When multiple alternative constraints are defined for the same candidate, the candidate is considered feasible if at least one alternative constraint is satisfied (OR).

The JSON database serves only as a repository of ontology-defined constraints. It does not perform ontology reasoning, generate semantic labels, or predict point annotations. The retrieved constraints are evaluated using the spatial information of the corresponding reference part during ontology-based feasibility refinement.

The organization and retrieval of the ontology constraints are illustrated in Figure 7.

**Reference Part Spatial Range Estimation.** Ontology constraints evaluate candidate feasibility using spatial relationships defined with respect to a reference part. Therefore, the spatial range of the ontology-defined reference part is estimated from the input point cloud as follows:

(11) $$R_{\mathrm{ref}}=SpatialRange\left(\mathrm{reference}\;\mathrm{part}\right).$$

The reference parts for Airplane, Knife, and Skateboard are *Body*, *Blade*, and *Deck*, respectively. The spatial range is estimated in the original coordinate system of the normalized point cloud, where front/back, up/down, and right/left correspond to the positive/negative *x*-, *y*-, and *z*-directions, respectively.

For Airplane, the point cloud is sliced along the *x*-axis to localize the *Body* based on the geometric distribution of each slice. The estimated $z_{\min}$ and $z_{\max}$ boundaries of the Body are used to evaluate the left/right spatial relationships of other parts.

For Skateboard, the *Deck* is localized using the center slice along the *x*-axis. Its estimated spatial range serves as the reference for evaluating the relative positions of other parts, including the *Wheel* and *Truck*.

For Knife, the point cloud is sliced along the *y*-axis to detect structural transitions associated with the guard using cross-sectional geometric characteristics. Because the guard is not represented as a separate semantic label, its estimated position is used as an auxiliary cue for determining the *Blade* reference range. Unlike Airplane and Skateboard, the Knife reference range is estimated separately for each slice, denoted by $R_{\mathrm{ref}}^{\left(s\right)}$, and the corresponding range is used when evaluating points within slice *s*.

If guard detection is unsuccessful, the spatial reference is considered unreliable and ontology-based filtering is skipped:

(12) $$L_{\mathrm{feas}}\left(x_{i}\right)=L_{\mathrm{sem}}\left(x_{i}\right).$$

This prevents uncertain guard detection from incorrectly eliminating plausible candidate interpretations.

Figure 8 illustrates the reference part localization for each object category and the category-specific spatial information used for ontology constraint evaluation.

**Ontology Constraint Evaluation.** For each candidate label $l\in L_{\mathrm{sem}}\left(x_{i}\right)$, the corresponding ontology constraints C(l) are retrieved from the constraint database. If no constraint is defined for a candidate, the candidate is retained, because the absence of a constraint is not considered evidence of infeasibility.

Each directional condition is evaluated using the estimated spatial range of the reference part and the coordinate system defined in Section 4.3. Directional relations require strict spatial separation from the corresponding reference-part boundary. For example, a point satisfies the right relation only when its *z*-coordinate exceeds the right-side boundary of the reference part. Points located exactly on the boundary do not satisfy the corresponding directional relation.

When a constraint contains multiple directional conditions, all conditions must be satisfied (AND). When multiple alternative constraints are defined for the same candidate, satisfying at least one constraint is sufficient (OR). Accordingly, the constraint evaluation function is defined as

(13) $$\Psi_{\mathrm{ont}}\left(x_{i},l\right)=\left\{\begin{array}{cc} 1, & C(l)=\emptyset ,\\ 1, & \exists c\in C(l)\;\mathrm{such}\;\mathrm{that}\;\mathrm{all}\;\mathrm{conditions}\;\mathrm{in}\;c\;\mathrm{are}\;\mathrm{satisfied},\\ 0, & \mathrm{otherwise}. \end{array}\right.$$

The feasible interpretation set is then constructed as

(14) $$L_{\mathrm{feas}}\left(x_{i}\right)=\left\{l\in L_{\mathrm{sem}}\left(x_{i}\right)|\Psi_{\mathrm{ont}}\left(x_{i},l\right)=1\right\}.$$

The point-wise implementation of this constraint evaluation and candidate filtering procedure is summarized in Algorithm 2.
**Algorithm 2:** Ontology-based Feasibility Refinement**Input:** Semantic interpretation sets ${\left\{L_{\mathrm{sem}}\left(x_{i}\right)\right\}}_{i=1}^{N}$; ontology constraint database *O*; object category *q*; reference part spatial range $R_{\mathrm{ref}}$; guard detection status *g* (for Knife)**Output:** Feasible interpretation sets ${\left\{L_{\mathrm{feas}}\left(x_{i}\right)\right\}}_{i=1}^{N}$$L_{\mathrm{feas}}\left(x_{i}\right)\leftarrow L_{\mathrm{sem}}\left(x_{i}\right)$ for all $x_{i}\in P;$


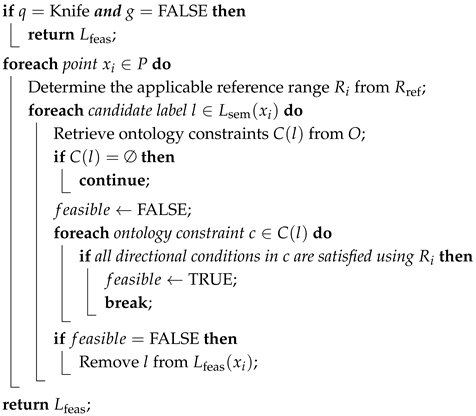



For example, given $L_{\mathrm{sem}}\left(x_{i}\right)=\left\{\mathrm{Wing},\mathrm{Body}\right\}$ for an Airplane point, the Wing candidate is retained only if it satisfies the applicable left/right spatial constraint relative to the Body reference part. Otherwise, it is removed.

Multiple candidates may remain feasible, whereas $L_{\mathrm{feas}}\left(x_{i}\right)=\emptyset$ when no candidate satisfies the applicable constraints. In either case, the ontology does not generate new labels or infer replacement labels.

For Knife, the same evaluation is performed using the slice-wise reference spatial range $R_{\mathrm{ref}}^{\left(s\right)}$ corresponding to each point. This incorporates the geometric information associated with the guard without introducing an additional semantic label.

Figure 9 illustrates the evaluation of ontology constraints for candidate filtering.

### 4.4. Overall Refinement Procedure

The overall procedure constructs $L_{\mathrm{sem}}\left(x_{i}\right)$ from the self-including 20-nearest-neighbor labels and evaluates each candidate against the category-specific ontology constraints and estimated reference-part range, as detailed in Section 4.2 and Section 4.3. For Knife, the slice-wise reference range $R_{\mathrm{ref}}^{\left(s\right)}$ is used when guard detection succeeds; otherwise, filtering is skipped and the semantic interpretation sets remain unchanged.

The resulting feasible interpretation set satisfies

(15) $$L_{\mathrm{feas}}\left(x_{i}\right)\subseteq L_{\mathrm{sem}}\left(x_{i}\right).$$

Depending on the applicable constraints, the result may retain one candidate, preserve multiple candidates, or be empty. No new or replacement label is inferred. Algorithm 2 summarizes the point-wise implementation.

In Algorithm 2, $R_{i}=R_{\mathrm{ref}}$ for Airplane and Skateboard, and $R_{i}=R_{\mathrm{ref}}^{\left(s\right)}$ for Knife when guard detection succeeds, where *s* is the slice containing $x_{i}$.

## 5. Experiment

In this section, we quantitatively and qualitatively evaluate the proposed ontology constraint-based annotation refinement framework. The goal of this study is not to directly modify the existing annotation and determine a single label but to selectively refine the semantic interpretation set constructed from local geometric context using ontology-defined structural and spatial constraints. Accordingly, we compare the changes between $L_{\mathrm{sem}}$ and $L_{\mathrm{feas}}$ to analyze the effect of ontology-based candidate filtering and examine how candidates are filtered by ontology constraints from both quantitative and qualitative perspectives.

### 5.1. Experimental Settings

**Dataset.** We conducted experiments using the test set of the ShapeNet Part Dataset. This choice allows us to examine annotation ambiguity independently of the data used for model training. ShapeNet Part is a representative 3D part segmentation benchmark that provides object-level 3D point clouds and point-wise part annotations [7]. We selected three categories, Airplane, Knife, and Skateboard, to examine ontology-based refinement across objects with different semantic and structural characteristics.

For Airplane, we focused on the Wing to examine ambiguity near the wing–body boundary and the feasibility of candidate labels under the adopted spatial constraints. This focus provides a representative case for analyzing spatial relationships between parts and does not imply that annotation ambiguity is limited to the Wing or its boundary.

For Knife, we considered label set incompleteness because the guard is not represented as an independent label in ShapeNet Part. Guard detection was performed for all 80 Knife objects, and ontology-based refinement was applied only to the 28 objects with detected guards; for the remaining 52 objects, $L_{\mathrm{feas}}=L_{\mathrm{sem}}$ was used.

For Skateboard, we considered ambiguity associated with the structural organization and spatial relationships of the deck, truck, and wheel.

These categories provide examples of different structural characteristics and annotation conditions rather than a one-to-one correspondence between object categories and the three sources of annotation ambiguity. Boundary ambiguity, part definition ambiguity, and label set incompleteness are conceptual sources that may coexist within the same object category.

In total, 341 Airplane, 80 Knife, and 31 Skateboard objects were analyzed, with 2048 points per object and no manual annotation correction.

**Ontology Configuration.** As described in Section 4.3, the ontology was constructed in OWL using Protégé [20,33] and converted into a JSON-based constraint database. Body, Blade, and Deck were used as the reference parts for Airplane, Knife, and Skateboard, respectively. During refinement, the database was used to retrieve the category- and candidate-specific structural and spatial constraints. For Knife, the detected guard was used only as a geometric cue for estimating the reference part spatial range. Ontology reasoning was not performed during point-level refinement; the retrieved constraints were directly evaluated against the estimated reference range.

**Implementation Details.** The semantic interpretation set was constructed using a *k*-nearest neighbor (k-NN) approach, and the unique labels observed within each neighborhood were retained as candidates. Ontology-based refinement then evaluated the corresponding structural and spatial constraints and retained feasible candidates. For Knife, guard detection was incorporated into reference part range estimation; when guard detection failed, $L_{\mathrm{feas}}=L_{\mathrm{sem}}$ was used. All experiments were implemented in Python 3.12.9 using NumPy 2.2.3, Pandas 2.2.3, scikit-learn 1.7.2, and Open3D 0.19.0. The experiments were conducted on a system with Intel Core i3-7100U CPU @ 2.40GHz and 8 GB RAM.

**Parameter Settings.** In all experiments, k=20 was used to construct the local semantic interpretation set, including the query point itself. No category-specific parameter tuning was performed during the reported experiments. The same refinement procedure was applied across all categories using their predefined ontology constraints and category-specific reference part localization procedures.

**Comparison Protocol.** To characterize the effect of different filtering criteria on the same input, we compared ontology-based refinement with a local candidate-pruning baseline inspired by Candidate Label Set Pruning (CLSP) [19]. The baseline is referred to as *CLSP-inspired* because it adapts the candidate-pruning principle to the present point-cloud candidate sets rather than reproducing the full CLSP learning algorithm. Both methods received the same $L_{\mathrm{sem}}\left(x_{i}\right)$ constructed from the original annotations. For the baseline, 20 nearest neighboring points excluding the query point were used to count, for each candidate, the neighboring sets in which that candidate was absent (down-votes). With pruning proportion τ=0.2, candidates with down-vote counts strictly above the rank-based pruning threshold were removed; ties at the threshold were retained. The procedure retained at least one candidate at every point. Unlike the ontology-based method, this baseline did not evaluate explicit part-to-part spatial constraints. The comparison covered the same 341 Airplane, 80 Knife, and 31 Skateboard test objects. We compared OFR, ER, PFR, and UR, point-wise disagreement between final candidate sets, and Wing candidate membership in two Airplane examples. This protocol evaluates candidate-set filtering behavior, not annotation correctness or predictive accuracy.

### 5.2. Evaluation Metrics

We evaluate candidate-set structure using AS and MLR, candidate removal using OFR, and point-level outcomes using UR, PFR, and ER. Object-wise reductions in AS and MLR support the distribution analysis.

**Ambiguity Size (AS).** Ambiguity Size (AS) represents the average number of semantic interpretation candidates associated with a point. In this study, it is defined separately for the semantic interpretation set and the feasible interpretation set as follows.

(16) $$AS_{\mathrm{sem}}=\frac{1}{N}\sum_{i=1}^{N}\left|L_{\mathrm{sem}}\left(x_{i}\right)\right|$$

(17) $$AS_{\mathrm{feas}}=\frac{1}{N}\sum_{i=1}^{N}\left|L_{\mathrm{feas}}\left(x_{i}\right)\right|$$

where *N* denotes the total number of points. $AS_{\mathrm{sem}}$ represents the average number of semantic interpretation candidates constructed from the local geometric context before ontology filtering, while $AS_{\mathrm{feas}}$ represents the average number of feasible interpretation candidates remaining after applying the ontology constraints.

A reduction in AS indicates that the average number of candidates per point has decreased. However, a reduction in AS itself is not interpreted as evidence that ambiguity has been resolved. In particular, AS can also decrease when all candidates are removed at some points by the ontology constraints, resulting in $L_{\mathrm{feas}}\left(x_{i}\right)=\emptyset$. Therefore, AS is interpreted together with MLR and ER to distinguish changes in candidate set size from the occurrence of empty feasible sets.

**Multi-label Ratio (MLR).** Multi-label Ratio (MLR) represents the proportion of points that have two or more semantic interpretation candidates among all points. It is defined as follows.

(18) $$MLR_{\mathrm{sem}}=\frac{1}{N}\sum_{i=1}^{N}I\left(|L_{\mathrm{sem}}\left(x_{i}\right)|\;>1\right)$$

(19) $$MLR_{\mathrm{feas}}=\frac{1}{N}\sum_{i=1}^{N}I\left(|L_{\mathrm{feas}}\left(x_{i}\right)|\;>1\right)$$

where I(·) is an indicator function that returns 1 when the condition is true and 0 otherwise.

MLR measures the proportion of points with two or more interpretation candidates rather than the number of candidates itself. Thus, a point with two candidates and a point with three candidates are both treated as multi-label points in MLR. In addition, a point with $L_{\mathrm{feas}}\left(x_{i}\right)=\emptyset$ has $|L_{\mathrm{feas}}\left(x_{i}\right)|\;=0$ and is therefore not counted as a multi-label point in MLR. Accordingly, reductions in MLR should also be interpreted together with the occurrence of empty feasible sets.

**Empty Ratio (ER).** Empty Ratio (ER) is an auxiliary metric representing the proportion of points for which no feasible interpretation remains after ontology-based refinement.

(20) $$ER=\frac{1}{N}\sum_{i=1}^{N}I\left(|L_{\mathrm{feas}}\left(x_{i}\right)|\;=0\right)$$

ER is used to examine whether reductions in AS and MLR are associated with selective reduction of feasible interpretations or are affected by the removal of all candidates at some points. Therefore, ER is not a metric for directly evaluating semantic correctness or accuracy of the refinement, but an auxiliary measure indicating the occurrence of points for which no feasible interpretation remains after refinement.

**Ontology Filtering Rate (OFR).** Ontology Filtering Rate (OFR) represents the proportion of semantic interpretation candidates removed during the ontology-based refinement process.

(21) $$OFR=\frac{\sum_{i=1}^{N}\left(|L_{\mathrm{sem}}\left(x_{i}\right)\left|\;-\;\right|L_{\mathrm{feas}}\left(x_{i}\right)|\right)}{\sum_{i=1}^{N}\left|L_{\mathrm{sem}}\left(x_{i}\right)\right|}$$

The numerator represents the total number of candidates removed during ontology-based refinement, while the denominator represents the total number of semantic interpretation candidates before refinement. Therefore, OFR satisfies 0≤OFR≤1.

An OFR of 0 indicates that no candidates were removed by the ontology constraints, while a higher OFR indicates that a larger proportion of the semantic interpretation candidates was filtered. However, a higher OFR does not necessarily indicate better refinement. Since OFR is a filtering measure representing the proportion of candidates removed by ontology-defined constraints, it is not interpreted as a direct measure of semantic correctness in this study.

**Point-level Filtering Behavior.** Although OFR represents the overall degree of candidate-level filtering, it does not distinguish how candidates are removed at individual points. To complement this measure, point-level filtering behavior after ontology refinement is divided into two cases, Unchanged and Partially Filtered, according to changes in the candidate set. Cases in which all candidates are removed are identified using the previously defined ER.

First, Unchanged Ratio (UR) represents the proportion of points for which the candidate set remains unchanged after refinement.

(22) $$UR=\frac{1}{N}\sum_{i=1}^{N}I\left(|L_{\mathrm{feas}}\left(x_{i}\right)\left|\;=\;\right|L_{\mathrm{sem}}\left(x_{i}\right)|\right)$$

In this study,

(23) $$L_{\mathrm{feas}}\left(x_{i}\right)\subseteq L_{\mathrm{sem}}\left(x_{i}\right)$$

Therefore, when the candidate set sizes are equal, the sets themselves are also identical. Thus, UR represents cases in which the candidate set at a point is not changed by the ontology constraints.

Partial Filtering Ratio (PFR) represents the proportion of points for which some candidates are removed while at least one feasible candidate remains.

(24) $$PFR=\frac{1}{N}\sum_{i=1}^{N}I\left(0<\;|L_{\mathrm{feas}}\left(x_{i}\right)\left|\;<\;\right|L_{\mathrm{sem}}\left(x_{i}\right)|\right)$$

For example,

(25) {Body,Wing}→{Wing}

represents a PFR case in which some candidates are removed while one or more feasible candidates remain. PFR represents the proportion of points where selective filtering occurs, such that incompatible candidates are removed by ontology constraints while feasible interpretations are retained. However, PFR does not represent semantic correctness or an ambiguity resolution rate; it represents a filtering outcome in which some candidates are removed and at least one candidate remains.

When all semantic candidates are removed and $L_{\mathrm{feas}}\left(x_{i}\right)=\emptyset$, the point is included in the previously defined ER. Thus, ER can also be used as an auxiliary measure representing the case in which all candidates are removed in the point-level filtering behavior.

**Figure 10:**
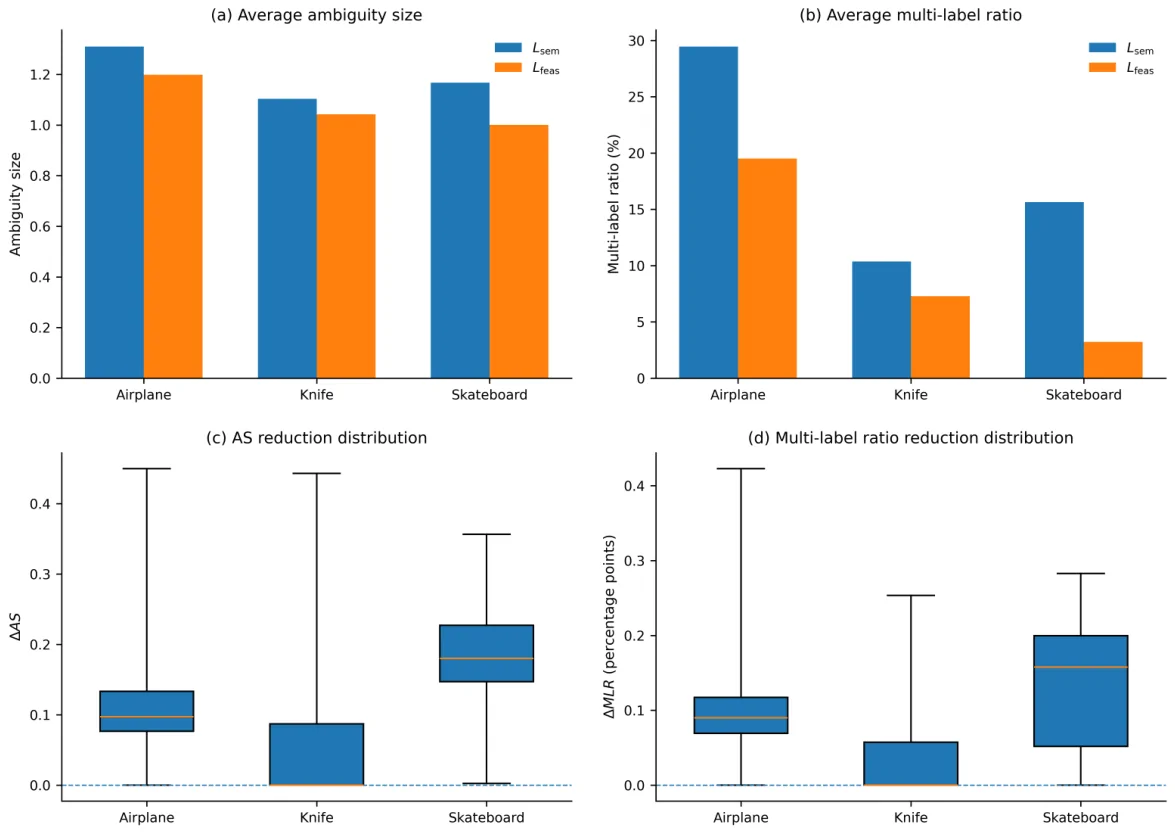
Ambiguity preservation analysis. (**a**) Category-wise comparison of average ambiguity size. (**b**) Category-wise comparison of average multi-label ratio. (**c**) Distribution of AS reduction across objects. (**d**) Distribution of multi-label ratio reduction across objects. In (**a**,**b**), blue and orange denote the semantic and feasible interpretation sets, respectively; in (**c**,**d**), the blue boxes show the interquartile ranges of object-wise reductions, the orange horizontal lines indicate the medians, the black whiskers show the spread beyond the quartiles, and the blue dotted horizontal lines indicate zero reduction.

**Reduction Measures.** To analyze the object-wise distributions in Figure 10, the differences in ambiguity size and multi-label ratio before and after refinement were calculated. For object *o*, the reduction in ambiguity size is defined as follows.

(26) $$\Delta AS_{o}=AS_{\mathrm{sem},o}-AS_{\mathrm{feas},o}$$

The absolute reduction in multi-label ratio is defined as follows.

(27) $$\Delta MLR_{o}=MLR_{\mathrm{sem},o}-MLR_{\mathrm{feas},o}$$

In this study, the reduction in ambiguity size in Figure 10c was calculated as $\Delta AS_{o}$, and the reduction in multi-label ratio in Figure 10d was calculated as $\Delta MLR_{o}$ to compare object-wise distributions. These reduction measures are descriptive measures representing changes in candidate set size observed after refinement and do not represent semantic correctness or an ambiguity resolution rate. The evaluation metrics used in this study are summarized in Table 2.

The object-wise reductions $\Delta AS_{o}$ and $\Delta MLR_{o}$ are used for the distributions in Figure 10; none of these metrics independently establishes semantic correctness.

### 5.3. Ambiguity Preservation Analysis

This section examines changes from $L_{\mathrm{sem}}\left(x_{i}\right)$ to $L_{\mathrm{feas}}\left(x_{i}\right)$ using AS and MLR, with ER indicating points where no feasible candidate remains.

Table 3 presents the average ambiguity statistics before and after ontology-based refinement for the three object categories. Since $L_{\mathrm{feas}}\left(x_{i}\right)\subseteq L_{\mathrm{sem}}\left(x_{i}\right)$ by construction, AS and MLR cannot increase after filtering. Accordingly, the observed reductions in both metrics are inherent consequences of the candidate-filtering operation rather than independent evidence of ambiguity resolution or improved annotation accuracy. The category-wise values characterize the extent of candidate-set reduction under the adopted ontology constraints.

The largest reductions in both metrics occurred for Skateboard (Figure 10a,b). Because empty sets also lower AS and are excluded from multi-label counts, these reductions must be interpreted alongside ER rather than as evidence that valid interpretations were preserved.

Table 4 shows that ER remained below 3.1% in every category, indicating that most points retained at least one feasible interpretation. This helps distinguish candidate reduction from wholesale removal of all candidates.

Object-wise reductions vary within each category (Figure 10c,d): many Skateboard objects exhibit relatively large MLR reductions, whereas some Knife objects show little change. Differences in local candidate composition and applicable structural constraints may contribute to this variation. These distributions describe filtering behavior, not independently verified semantic correctness.

### 5.4. Ontology Filtering Analysis

This section uses OFR to quantify candidate removal and UR, PFR, and ER to distinguish point-level filtering outcomes.

Table 5 shows that OFR was highest for Skateboard and lowest for Knife, with a macro-average of 0.0947 across the three categories.

OFR does not distinguish partial from complete candidate removal at individual points; Table 6 provides this distinction through UR, PFR, and ER.

Table 6 shows that Knife had the highest UR, with similar PFR and ER, whereas Skateboard had the lowest UR and highest PFR. Airplane filtering was predominantly partial when candidate sets changed.

UR, PFR, and ER represent cases in which the candidate set remains unchanged, some candidates are removed, and all candidates are removed, respectively. They are therefore mutually exclusive and partition all points. Thus,

(28) UR+PFR+ER=1

is satisfied. The sum was 1 for each category and the macro-average within rounding error. The macro-averages show that unchanged sets predominate. PFR and ER describe filtering outcomes, not semantic correctness; ER may also signal limitations in candidate construction, reference localization, or ontology constraints.

### 5.5. Category-Wise Discussion

We interpret category-level differences in terms of candidate composition and part structure.

**Airplane**

Airplane showed relatively limited filtering, with multiple candidates remaining after refinement. This behavior is consistent with wing–body boundary regions where multiple interpretations may remain compatible with the structural and spatial constraints. Thus, ontology filtering selectively restricts structurally inconsistent candidates rather than uniformly eliminating boundary ambiguity.

**Knife**

Knife showed the lowest overall filtering rate among the three categories. However, this result should be interpreted in the context of the guard-based processing used for this category, in which ontology-based filtering was applied only to the 28 objects for which guard detection was successful, while the remaining 52 objects were processed without ontology filtering. The guard was used as a spatial cue for estimating the Blade reference range rather than as a semantic candidate. The resulting behavior indicates that feasibility evaluation can still be performed under label set incompleteness without introducing the missing component as a new label.

Table 7 compares the results for all 80 Knife objects with those for the 28 objects in which guard regions were detected. Each object contains 2048 points. Ontology-based filtering was applied to the 28 guard-detected objects, whereas the semantic candidate sets of the remaining 52 objects were retained unchanged.

For the 28-object subset, AS changed from 1.0880 to 0.9129, while MLR changed from 0.0880 to 0.0001. The subset exhibited an OFR of 0.1586, with UR, PFR, and ER values of 82.49%, 8.79%, and 8.72%, respectively. The higher PFR and ER values relative to the complete Knife dataset indicate that candidate filtering was concentrated in the guard-detected subset. In particular, the relatively high ER shows that empty feasible sets contributed to the observed reduction in AS. These results characterize candidate removal under the adopted ontology constraints rather than demonstrating improved annotation accuracy.

**Skateboard**

Skateboard showed the highest OFR and PFR, indicating more frequent partial candidate filtering. This behavior is consistent with the structural organization of the deck, truck, and wheel, which provides spatial constraints for distinguishing locally ambiguous candidates. Nevertheless, compatible interpretations were preserved rather than being forced into a single label.

Filtering therefore varies with local candidates and category-specific structural constraints.

### 5.6. Qualitative Analysis

**Figure 11:**
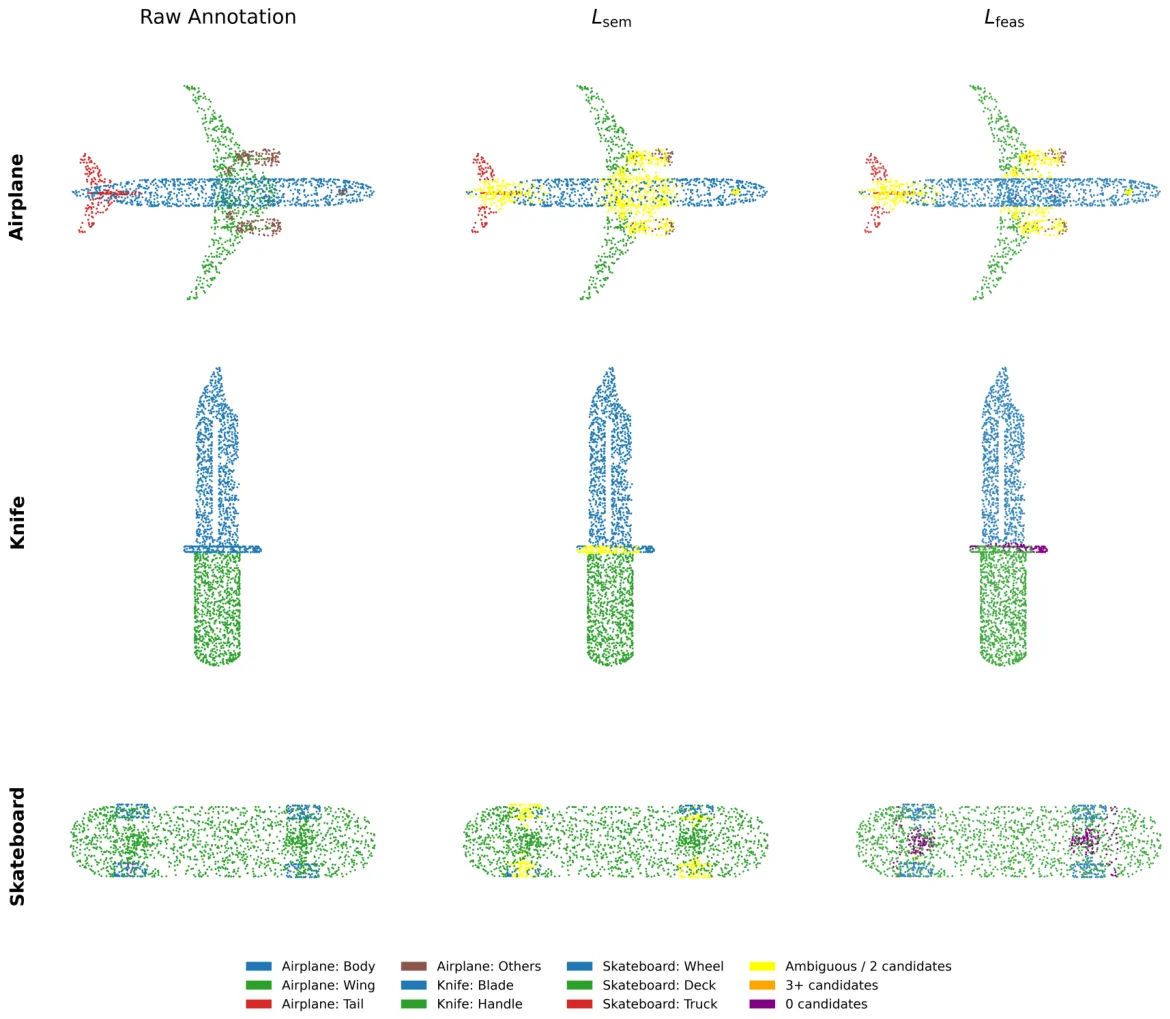
Qualitative examples of ontology-based ambiguity refinement for Airplane, Knife, and Skateboard. For each category, Raw Annotation, semantic interpretation set $L_{\mathrm{sem}}$, and feasible interpretation set $L_{\mathrm{feas}}$ are compared. Yellow, orange, and purple indicate points with two, three or more, and zero candidates, respectively. The same candidate-count-based color scheme is applied to both $L_{\mathrm{sem}}$ and $L_{\mathrm{feas}}$. The examples illustrate that ontology-compatible ambiguity can be preserved, while candidates incompatible with ontology-defined structural and spatial constraints can be selectively removed.

Figure 11 compares Raw Annotation, $L_{\mathrm{sem}}$, and $L_{\mathrm{feas}}$ for representative Airplane, Knife, and Skateboard examples to illustrate point-level filtering outcomes.

**Airplane: Selective Filtering and Ambiguity Preservation**

In the Airplane example, local geometric context produces candidates such as $L_{\mathrm{sem}}\left(x_{i}\right)=\left\{Body,Wing\right\}$. At some points, the Wing candidate is incompatible with the ontology constraints and is removed, yielding {Body}. At actual wing–body boundary regions, however, both candidates may remain feasible, as in {Body,Wing}→{Body,Wing}. This demonstrates that the same candidate set can lead to different outcomes depending on structural and spatial context.

**Knife: Ontology-guided Refinement under Label Set Incompleteness**

In the Knife example, ambiguity near the Blade–Handle boundary can produce {Blade,Handle}→{Handle} after ontology filtering. In another region, the current candidate set may contain only {Blade}, which can become infeasible and result in {Blade}→∅. The guard is used only as a spatial cue for evaluating the existing candidates and is not introduced as a new label.

**Skateboard: Part-Label Mismatch and Feasibility-based Candidate Filtering**

In the Skateboard example, the physical structure contains Deck, Truck, and Wheel, while part of the Truck region is annotated as Deck. We therefore describe this case as an annotation–structure mismatch rather than treating it as a ground-truth correction. Near the Wheel–Deck boundary, the candidate set may change from {Wheel,Deck} to {Wheel} when the Deck candidate is incompatible with the spatial constraints. In contrast, a region represented only by the Deck candidate may result in an empty feasible set when that candidate is structurally incompatible.

### 5.7. Comparison with Local Candidate-Frequency-Based Filtering

To examine the effect of the filtering criterion independently of candidate-set construction, we applied ontology-based refinement and the CLSP-inspired baseline to the same semantic interpretation sets. Table 8 compares their filtering behavior across the three object categories.

The methods exhibited different filtering patterns across categories. For Airplane, the CLSP-inspired baseline had a slightly higher OFR than ontology-based refinement (9.87% versus 8.53%), but a substantially higher PFR (20.20% versus 10.77%). For Knife and Skateboard, ontology-based refinement had higher OFR values. Thus, the overall proportion of removed candidates does not by itself characterize the distribution of point-level filtering outcomes. The baseline produced no empty sets because its pruning rule retained at least one candidate, whereas ontology-based refinement permitted empty feasible sets when no candidate satisfied the adopted constraints. This difference follows from the methods’ definitions and is not evidence of relative annotation correctness.

We further compared the final candidate sets at each point, counting a disagreement whenever the sets were unequal. Table 9 summarizes the results.

Across all 925,696 points, the two methods yielded different candidate sets at 150,882 points (16.30%). Of these, 37,507 points were changed only by ontology-based filtering, 98,205 only by CLSP-inspired filtering, and 15,170 by both methods but with different final candidate sets. These counts quantify differences in filtering decisions without determining which candidate composition is semantically correct.

**Figure 12:**
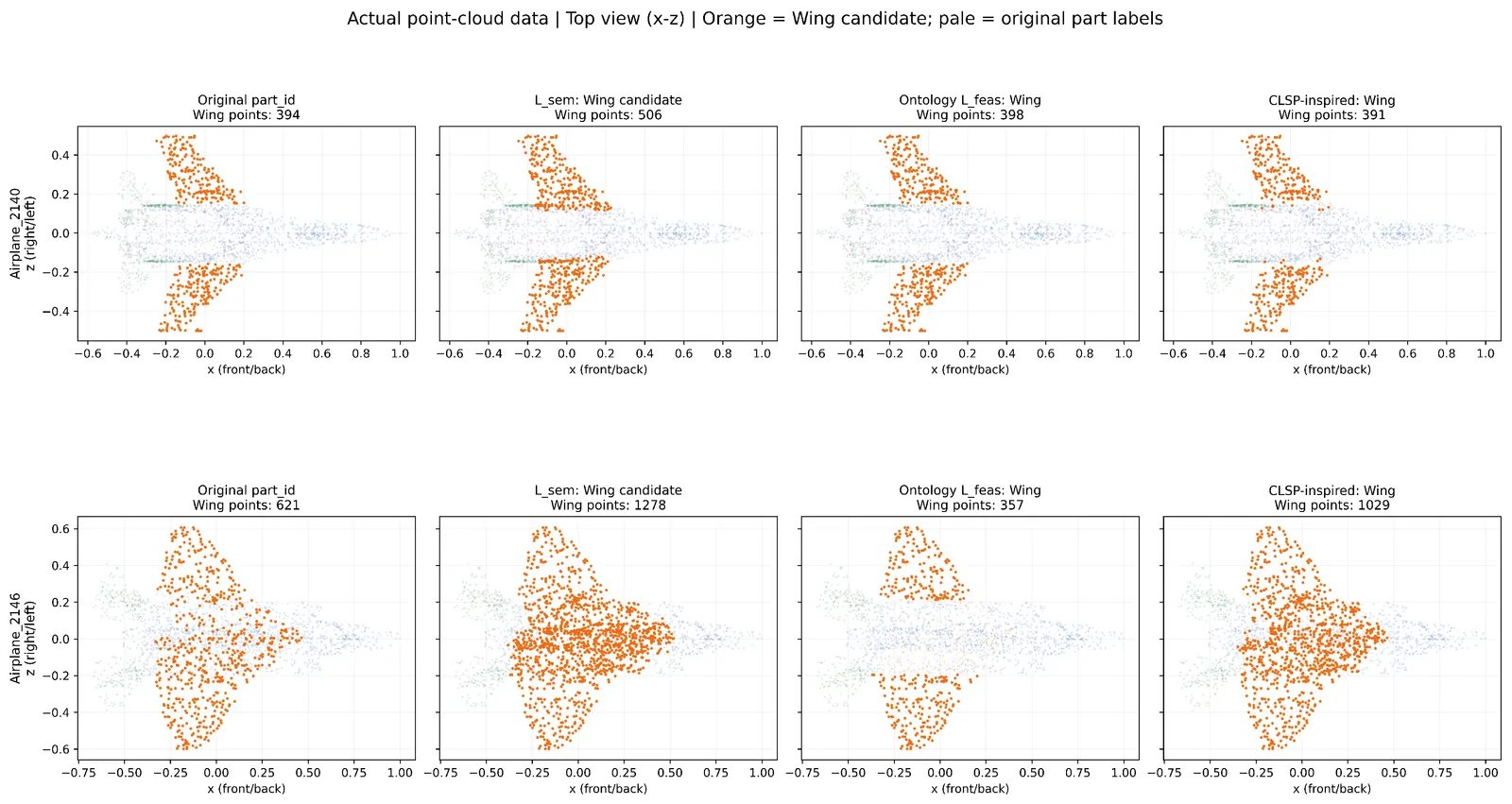
Top-view comparison of Wing candidate membership for Airplane_2140 and Airplane_2146. The columns show original point-wise annotations, initial semantic interpretation sets, ontology-based filtering, and CLSP-inspired filtering. Orange points indicate points annotated as Wing in the original single-label annotation (first column) or points whose candidate sets contain Wing in the remaining columns. Pale colors show the original part labels of the other points for visual context; Wing candidate membership in the remaining columns does not imply a single-label Wing assignment.

Figure 12 provides a spatial comparison of Wing candidate membership in Airplane_2140 and Airplane_2146. Wing candidate membership denotes the presence of Wing in a point’s candidate set; it is not equivalent to assigning Wing as that point’s single annotation. The initial semantic interpretation sets contain more Wing candidates than the original point-wise Wing annotations because $L_{\mathrm{sem}}\left(x_{i}\right)$ contains all unique labels observed within the self-including 20-nearest-neighbor neighborhood. Accordingly, points originally annotated as Body may also contain Wing as a candidate when Wing-labeled neighbors are present. This expansion represents locally observed alternatives, not newly predicted or reassigned labels.

In Airplane_2140, Wing appeared in the initial semantic candidate sets at 506 points, compared with 398 points after ontology-based filtering and 391 after CLSP-inspired filtering. In Airplane_2146, the corresponding counts were 1278, 357, and 1029. Although the retained counts were similar in the first example, the second exhibited a substantial difference: many Wing candidates in the central body region remained under local candidate-based pruning but were removed by the ontology-based method according to the adopted left/right relationship between Wing and Body. Removing Wing membership from a candidate set does not imply that the point was deleted or that its annotation was reassigned.

The visual comparison illustrates the different information used for filtering: local candidate distributions in the CLSP-inspired baseline versus explicit spatial and structural constraints in the proposed method. In particular, the Airplane_2146 result demonstrates constraint-based removal of Wing candidates that are incompatible with the adopted ontology. These observations establish differences in candidate-set refinement behavior, not independently verified semantic correctness or superiority over the baseline.

## 6. Discussion

### 6.1. Ambiguity Preservation and Filtering Behavior

The observed changes in candidate sets should be interpreted as selective feasibility filtering, not as independent evidence of ambiguity resolution.

The point-level analysis further distinguishes unchanged candidate sets, partial filtering, and empty feasible sets. PFR and ER distinguish partial removal from cases with no feasible candidate; neither establishes semantic correctness.

**Category-dependent Refinement Behavior.** Filtering behavior depends on the selected category-specific constraints and the availability of usable reference structures, as illustrated by the Knife guard-detection subset.

### 6.2. Empty Feasible Sets and Methodological Limitations

An empty feasible set, $L_{\mathrm{feas}}\left(x_{i}\right)=\emptyset$, indicates that none of the current semantic candidates satisfies the applicable ontology constraints. It does not imply that a replacement label has been inferred or that the original annotation has been corrected.

Such cases may arise from limitations in local candidate construction, reference part localization, ontology constraint representation, or the predefined label vocabulary. For example, the Knife guard is not represented as an independent semantic label in ShapeNet Part; its geometric information is used only as a spatial cue. Consequently, the framework cannot introduce Guard as an additional candidate when the existing vocabulary is insufficient.

The present evaluation characterizes candidate-set changes and filtering behavior using AS, MLR, ER, OFR, UR, and PFR. These measures do not directly establish agreement with ground-truth semantic labels. Therefore, the results should not be interpreted as demonstrating an absolute improvement in annotation accuracy.

As summarized in Table 1, the representative approaches differ from the proposed framework in their research objectives, principal mechanisms, and output representations. Direct quantitative benchmarking against those original methods was not performed because their existing evaluation protocols and output representations do not directly assess the candidate-level feasibility and preservation behavior considered here. Instead, Section 5.7 provides a controlled comparison with a CLSP-inspired candidate-pruning baseline under a common initial candidate-set representation. This limited comparison characterizes differences in filtering decisions; it neither reproduces the full CLSP algorithm nor establishes comparative annotation accuracy. A broader performance comparison would require a common evaluation protocol and independent criteria for semantic correctness and the preservation of plausible interpretations.

### 6.3. Interpretation of the Filtering Comparison

The comparison with CLSP-inspired filtering provides a complementary view of the proposed framework’s behavior under the same initial semantic interpretation sets. The two methods use different information for candidate removal: the baseline relies on local candidate distributions, whereas the proposed framework evaluates ontology-defined structural and spatial constraints. Their differing OFR, PFR, UR, and ER values, together with point-wise disagreement, characterize how these filtering criteria affect candidate-set composition rather than ranking the methods by annotation quality.

The Airplane_2146 example illustrates the interpretive limit of this difference: explicit Body–Wing constraints can reject candidates retained by local-frequency pruning, but their feasibility remains conditional on the adopted Wing definition. The example does not independently establish semantic correctness.

Because the adapted baseline retains at least one candidate whereas ontology filtering permits empty sets, the comparison cannot establish predictive superiority or annotation accuracy improvement.

### 6.4. Generalizability and Future Directions

The primary application scenario is the analysis and refinement of existing part-level annotations in object-level 3D point cloud datasets. The framework can support annotation quality assessment by identifying candidate interpretations that are incompatible with structural and spatial constraints while preserving feasible alternatives. Although the present experiments were conducted on the Airplane, Knife, and Skateboard categories of ShapeNet Part, the underlying refinement procedure is not inherently restricted to this dataset. In principle, it could be adapted to other part-annotated object datasets, such as PartNet-Mobility and datasets associated with SAPIEN, provided that suitable part labels, ontology constraints, and reference part localization methods are available.

Extension to indoor and outdoor scene-level datasets, including S3DIS, ScanNet, SemanticKITTI, and Semantic3D, would require additional adaptation. These datasets primarily target scene-level semantic segmentation or related tasks rather than the object-specific part annotation setting considered in this study. Applying the proposed framework would therefore require identifying relevant object instances, establishing part-level candidate labels, and defining category-specific structural and spatial constraints with appropriate reference parts. For articulated objects or varying configurations, the ontology constraints may also need to account for changes in relative part positions.

The present results do not establish effectiveness on these additional datasets or broader object categories. Furthermore, the current framework evaluates candidate feasibility under a selected ontology configuration and does not simultaneously accommodate alternative, potentially incompatible part definitions, such as different structural definitions of an airplane Wing. Supporting multiple ontology configurations or alternative part definitions within a unified refinement procedure remains an important direction for future research. In addition, the semantic interpretation sets were constructed using a fixed neighborhood size of k=20, without a systematic sensitivity analysis. Future work should investigate adaptation to more diverse object and scene datasets, neighborhood-size sensitivity, richer structural constraints, and uncertainty in reference part localization. Ontology expansion, part discovery, and independent semantic validation, including human annotation agreement, also remain important directions for evaluating annotation quality beyond candidate filtering behavior.

## 7. Conclusions

In this study, we proposed an ontology-based annotation refinement framework that does not simply regard semantic ambiguity arising in 3D part annotation as annotation error. Instead, the proposed framework explicitly represents possible semantic interpretations and selectively restricts candidates that are incompatible with ontology-defined structural and spatial constraints. Rather than directly modifying the existing annotation or forcing a single label, the framework first constructs a semantic interpretation set $L_{\mathrm{sem}}\left(x_{i}\right)$ for each point based on annotation labels observed in its local neighborhood, and then obtains the feasible interpretation set $L_{\mathrm{feas}}\left(x_{i}\right)$ by filtering these candidates using ontology constraints.

Since the feasible interpretation set is constructed as a subset of the semantic interpretation set, the non-increasing behavior of AS and MLR is an inherent property of ontology-based filtering rather than an independent empirical finding. The experiments on Airplane, Knife, and Skateboard from ShapeNet Part characterize the extent and distribution of candidate filtering under the adopted ontology constraints. The macro-average OFR was 9.47%, indicating the average proportion of semantic candidates removed by ontology-based filtering. The point-level analysis showed that candidate sets remained unchanged for a macro-average of 88.93% of points, while 8.89% exhibited partial filtering, in which some candidates were removed while at least one feasible interpretation was retained. These results describe how ontology constraints affect candidate sets without establishing improved annotation accuracy or semantic correctness.

Meanwhile, all candidates were removed at a macro-average of 2.18% of points, resulting in $L_{\mathrm{feas}}\left(x_{i}\right)=\emptyset$. Such an empty feasible set indicates that no interpretation in the current candidate set is compatible with the ontology constraints and does not imply that a new semantic label was generated or that the corresponding annotation was directly corrected. Therefore, this outcome is interpreted as a diagnostic behavior associated with limitations in local semantic candidate generation, reference part spatial range estimation, ontology constraint representation, and related components.

Future work should investigate broader object categories and part hierarchies, uncertainty in reference part localization, and ways to represent semantic components absent from predefined label sets. Independent semantic validation will also be needed to assess annotation quality beyond the observed candidate-filtering behavior.

## Figures and Tables

**Table 1:** Comparison of research approaches to annotation refinement and semantic reasoning.

Approach	Research Objective	Principal Mechanism	Output Focus
PNAL [16]	Robust point-cloud segmentation with noisy labels	Confidence selection and cluster-wise label correction	Corrected supervision and segmentation
CLSP [19]	Candidate pruning in partial-label learning	Representation–candidate space inconsistency	Pruned candidate sets
Codiglione et al. [31]	Multi-granularity semantic representation	Ontology and compositional hierarchy	Multi-level semantic labels
Goldhoorn and Hartanto [32]	Spatially constrained semantic labeling	Spatial object constraints	Semantic object labels
Proposed framework	Ambiguity-aware part annotation refinement	Local semantic candidate construction and ontology feasibility filtering	Feasible candidate sets

**Table 2:** Summary of evaluation metrics.

Metric	Measurement	Meaning
**AS**	Mean number of candidates	Average size of the interpretation set per point
**MLR**	Ratio of multi-label points	Proportion of points exhibiting multiple candidates
**ER**	Ratio of empty feasible sets	Proportion of points with no feasible candidate
**OFR**	Ratio of removed candidates	Overall degree of candidate filtering
**UR**	Ratio of unchanged points	Proportion of points whose candidate sets remain unchanged
**PFR**	Ratio of partially filtered points	Proportion of points where some candidates are removed while at least one remains

**Table 3:** Average ambiguity statistics of the semantic interpretation set and feasible interpretation set for each object category.

	Semantic Interpretation Set		Feasible Interpretation Set	
Category	${\mathrm{AS}}_{\mathrm{sem}}$	${\mathrm{MLR}}_{\mathrm{sem}}$	${\mathrm{AS}}_{\mathrm{feas}}$	${\mathrm{MLR}}_{\mathrm{feas}}$
Airplane	1.3103	0.2946	1.1985	0.1951
Knife	1.1036	0.1036	1.0423	0.0728
Skateboard	1.1675	0.1563	1.0003	0.0322

**Table 4:** Empty Ratio (ER) after ontology-based refinement for each object category.

Category	ER
Airplane	0.4107%
Knife	3.0518%
Skateboard	3.0651%
Macro-average	2.1759%

**Table 5:** Ontology Filtering Rate (OFR) for each object category.

Category	OFR
Airplane	0.0853
Knife	0.0555
Skateboard	0.1433
Macro-average	0.0947

**Table 6:** Point-level filtering behavior after ontology-based refinement.

Category	UR	PFR	ER
Airplane	88.8234%	10.7660%	0.4107%
Knife	93.8727%	3.0756%	3.0518%
Skateboard	84.1009%	12.8339%	3.0651%
Macro-average	88.9323%	8.8918%	2.1759%

**Table 7:** Comparison of ontology-based filtering statistics for all Knife objects and the subset with detected guard regions.

Metric	All Knife Objects (N=80)	Guard-Detected Objects (N=28)
Number of points	163,840	57,344
$AS_{\mathrm{sem}}$	1.1036	1.0880
$AS_{\mathrm{feas}}$	1.0423	0.9129
$MLR_{\mathrm{sem}}$	0.1036	0.0880
$MLR_{\mathrm{feas}}$	0.0728	0.0001
OFR	0.0555	0.1586
UR (%)	93.87	82.49
PFR (%)	3.08	8.79
ER (%)	3.05	8.72

**Table 8:** Category-wise comparison of candidate-set filtering behavior (%).

Category	Method	OFR	ER	PFR	UR
Airplane	Ontology-based	8.53	0.41	10.77	88.82
	CLSP-inspired	9.87	0.00	20.20	79.80
Knife	Ontology-based	5.55	3.05	3.08	93.87
	CLSP-inspired	3.04	0.00	6.09	93.91
Skateboard	Ontology-based	14.33	3.07	12.83	84.10
	CLSP-inspired	6.02	0.00	12.37	87.63

**Table 9:** Point-wise disagreement between ontology-based and CLSP-inspired candidate sets.

Category	Total Points	Disagreement Points	Disagreement (%)
Airplane	698,368	129,861	18.59
Knife	163,840	14,489	8.84
Skateboard	63,488	6532	10.29
Total	925,696	150,882	16.30

## Data Availability

The ShapeNet Part Dataset used in this study is publicly available through the PointNet project repository (https://github.com/charlesq34/pointnet/tree/master/part_seg (accessed on 4 November 2026)), which provides access instructions for the HDF5 dataset. The experiments were conducted using the test split (ply_data_test0.h5 and ply_data_test1.h5). The derived data and analysis results generated during this study are available from the corresponding author upon reasonable request.
